# Nutrition Management in Children Less than 5 Years of Age with Glycogen Storage Disease Type I: Survey Results

**DOI:** 10.3390/nu16193244

**Published:** 2024-09-25

**Authors:** Mary Sowa, Monica Boyer, Jessica Green, Surekha Pendyal, Heather Saavedra

**Affiliations:** 1 Division of Metabolics, Children’s Hospital of Orange County, Orange, CA 92868, USA; marysowa5@gmail.com; 2 Division of Medical Genetics, Department of Pediatrics, Duke University Medical Center, Durham, NC 27710, USA; jessica.green2@duke.edu (J.G.); surekha.pendyal@duke.edu (S.P.); 3 Division of Medical Genetics, Department of Pediatrics, McGovern Medical School at the University of Texas Health Science Center at Houston (UTHealth Houston), Children’s Memorial Hermann Hospital, Houston, TX 77030, USA; heather.saavedra@uth.tmc.edu

**Keywords:** GSD I, nutrition management, children, cornstarch

## Abstract

**Background:** Nutrition management for GSD Type I (GSDI; OMIM #232200, 232220) is complex, with the goal being to maintain euglycemia while minimizing metabolic derangements. Management guidelines were published in 2002 and 2014. However, there is limited information on the nuances of nutrition management and the unique feeding challenges of children. **Methods:** A REDCap survey focusing on staffing and current practices in the nutrition management of children with GSD I who were <5 years of age was sent to the metabolic dietitian’s listserv and GMDI membership in 8/2023. **Results:** There were 21 North American respondents. In 17/21 clinics (81%), Prosobee^®^ was the primary choice for infant formula. Dietitians used different methods to determine hourly glucose needs. Fasting recommendations ranged from 1 to 3 h, and the use of nighttime continuous feeding was common. Cornstarch was started between 6 and 12 months of age. Most clinics did not use Glycosade^®^ for children <5 years of age. Oral motor dysfunction, gagging, and lack of interest in food were common. Continuous glucose monitoring (CGM) devices were recommended in 20 clinics (95%). Most clinics followed patients on an outpatient basis. All clinics provided a hypoglycemia management plan; however, there was wide variability in practice. **Conclusion:** This survey highlights the variability in the care of individuals <5 years of age with GSD I. Updated guidelines are needed to help address the unique nutrition challenges in this age group.

## 1. Introduction

Glycogen storage disease types Ia and b (GSD Ia/b) are inherited autosomal recessive diseases caused by deficiencies in glucose-6-phosphatase (type Ia) and glucose-6-phosphate transporter gene (type Ib), leading to the malfunction of both glycogenolysis and gluconeogenesis [1,2]. These genetic defects also result in glycogen and fat accumulation in the liver and kidneys. Individuals with GSD Ia/b present with hepatomegaly, hypoglycemia, lactic acidosis, hyperuricemia, and hypertriglyceridemia. Without optimal treatment, individuals with GSD Ia/b may experience short stature, delayed puberty, renal disease, gout, systemic hypertension, pulmonary hypertension, hepatic adenomas, polycystic ovaries, and osteoporosis [1]. Individuals with GSD Ib also have impaired neutrophil function and increased susceptibility to infections and oral and intestinal mucosa ulcerations [1,2,3]. In 2019, the mechanism for neutropenia and neutrophil dysfunction was elucidated, which led to treatment with the SGLT2-inhibitor empagliflozin [4]. Off-label treatment with empagliflozin enhances the urinary excretion of the toxic metabolite 1,5-anhydroglucitol and improves neutrophil count and function [5].

Guidelines for the management of GSD Ia/b were published initially in 2002 by the European Study on Glycogen Storage Disease Type I [6] and most recently by GSD experts in the United States [1]. Although GSD Ia and Ib are different conditions, their nutrition management is similar, and both guidelines provide an overview of the nutritional care and medical management of both subtypes.

The goal of nutrition management for GSD Ia/b in young children is to maintain euglycemia and prevent or minimize lactic acidosis, hepatomegaly, hypertriglyceridemia, hyperuricemia, and failure to thrive [1]. Both guidelines address infant and toddler feeding practices. Table 1 details the recommendations from both guidelines.

In a more recent publication, the disparities and controversies between different guidelines and dietary practices were addressed, with recommendations for stricter control of dietary carbohydrates and sugars along with precision in uncooked cornstarch (UCCS) dosing [2]. An update in Gene Reviews^®^ provided broad guidance for using UCCS, alternating with frequent meals and snacks high in complex carbohydrates [3]. The restriction of fructose, sucrose, and galactose, which are non-utilizable sugars in GSD Ia/b, was also recommended to prevent lactic acidosis and storage in the liver [1,2,3]. Despite the guidance available at this time, the nuances of managing young children diagnosed with GSD Ia/b are lacking.

## 2. Materials and Methods

The authors developed a 44-item questionnaire with the goal of obtaining best practices in the care of individuals with GSD Ia/b <5 years of age at North American clinics. A copy of the survey and a summary of results are available in the Supplementary Data. The survey was sent to metabolic dietitians through the listserv (PNO-METAB-L@LISTSERV.CC.EMORY.EDU) and the Genetic Metabolic Dietitian International membership email list. Respondents had one month to complete the survey. The survey was created in REDCap, and responses were anonymous. We were unable to determine the estimated response rate, but 27 responses were received, and 6 international surveys were excluded from the summary. The quality improvement initiative was reviewed and determined to not meet the criteria for human subject research by the Children’s Hospital of Orange County Institutional Review Board. An abstract of the survey results was presented at the Genetic Metabolic Dietitians International Conference in April 2024 [7].

## 3. Results

### 3.1. Description of Care Team

Before answering the 44-item questionnaire, each respondent was asked to describe their clinic support. Table 2 describes the care team members in the 21 clinics. The support staff varied between clinics, but it included social workers, medical assistants, and other health team members. There were a few clinics with very large GSD populations, but on average, each clinic surveyed managed 11 individuals with GSD Ia/b, with 2 of these patients being <5 years old.

### 3.2. Formula Selection

Several questions were developed to assess formula use in this population. Formula selection is frequently based on the carbohydrate source and the content of the formula. Many acceptable formulas are elemental; however, not all patients require an elemental or semi-elemental formula. Table 3 reflects the most commonly prescribed infant and toddler formulas. There were several other formulas that fit the recommended criteria for formula selection but were used less frequently. For clinics that used continuous nighttime feedings, patients were transitioned to formulas with a higher carbohydrate content (i.e., Tolerex^®^), when they were older or to reduce caloric intake. There were two clinics (10%) that used UCCS instead of continuous feedings. Other common pediatric nutrition practices used in this population included concentrating formulas (14/21, 67%) to meet calorie or carbohydrate needs and, less often, a carbohydrate additive (maltodextrin, i.e., Polycal^®^; 8/21, 38%).

### 3.3. Tube Feedings

Gastrostomy tubes (GTs) are commonly used in the GSD Ia/b population due to the need for frequent and overnight feedings. Our survey found that seven clinics (33%) used GT feedings in all their patients, while nine other clinics (43%) used them in the majority of their patients. One clinic used nasogastric feedings instead of GT feedings. When asked if there were concerns about GT placement in type Ib, several clinics required an absolute neutrophil count of >500 for placement due to concerns about poor wound healing. To enhance the safety of nighttime feedings, nine clinics (43%) recommended bedwetting alarms to detect when a tube feeding was disconnected.

### 3.4. Glucose/Carbohydrate Requirement Estimation

Our survey found that 12/21 clinics (57%) used an estimated glucose production (EGP) rate to estimate carbohydrate requirements and calculate the same using the equation Y = 0.0014x^3^ − 9.214x^2^ + 10.411x − 9.084, where Y = mg/min glucose and X = ideal body weight in kg [8]. Ten survey recipients (48%) used the glucose infusion rate (GIR) to calculate carbohydrate requirements. Clinics followed glucose, lactate, and triglyceride levels to evaluate fasting tolerance. Twelve of the 21 clinics (57%) agreed that all three parameters (glucose, lactate, and triglycerides) are important when evaluating fasting tolerance, while the other clinics focused on glucose or lactate alone. Table 4 presents the clinics’ perceptions of the “average” fasting periods tolerated for selected age groups.

### 3.5. Initiating UCCS

UCCS has been used to improve fasting tolerance since the early 1980s; the ACMG guidelines recommend introducing UCCS between 6 and 12 months of age, while the European guidelines recommend its introduction after 1 year of age [1,6]. Table 5 indicates at what age clinics introduced UCCS in children with GSD Ia/b. Tapioca starch was recommended in 6/21 clinics (29%) if UCCS was not tolerated. The use of pancreatic enzymes was not common but was used in 5/21 clinics (24%) if a patient did not tolerate UCCS. Most clinics provided their recommendations for initiating UCCS to minimize gastrointestinal symptoms, such as bloating, gassiness, constipation, or diarrhea. The responses in the survey suggested beginning with small amounts (1 g or 0.5–1 tsp) every or every other feeding and advancing UCCS doses gradually every 2–3 day, until goal dosing was achieved.

In 2012, a waxy maize extended-release cornstarch, Glycosade^®^ a product by Vitaflo^®^ (Vitaflo International Ltd., Liverpool, UK), was approved for use in patients 5 years of age and older in the United States. Table 6 presents the use of Glycosade^®^ in the 21 clinics either during the nighttime and/or daytime. When initiating Glycosade^®^ treatment, 10/21 clinics (48%) admitted patients, while 8/21 (38%) initiated treatment in the home setting.

### 3.6. Oral Feedings of Solid Food

Table 7 provides an overview of “first” food groups recommended by dietitians for children with GSD Ia/b. Specific recommendations regarding the amount of carbohydrate (which ranged from 5 to 20 g in one meal) and simple sugar allowed per meal were provided by 13/19 (68%) and 3/19 clinics (16%), respectively. One clinic (5%) specified the portion size of food from each food group the infant should be offered at a meal. Five of nineteen clinics (26%) did not provide recommendations regarding carbohydrate content in solid foods due to limited intake at this age and infant feeding stage. Two clinics (10%) did not provide a response.

Table 8 indicates the most common barriers preventing normal feeding development in children with GSD Ia/b. Poor appetite, picky eating, and fullness related to frequent feedings required to maintain metabolic control were also mentioned as additional challenges in feeding young children with GSD Ia/b. Eighteen of twenty-one clinics (86%) referred their patients to feeding therapy to improve oral motor function.

### 3.7. Supplementation

Nutritional deficiencies are well-documented in GSD Ia/b, and supplementation with sugar-free vitamin and mineral supplements, vitamin D, calcium are considered important due to the restrictive nature of the diet and the use of UCCS, which provides substantial calories of poor nutritional value [9,10]. Table 9 reflects the respondents’ use of multivitamin and probiotic supplementation in individuals <5 years of age with GSD Ia/b.

### 3.8. Blood Glucose (BG), Continuous Glucose, and Lactate Monitoring

Finger-stick BG (FSBG) monitoring is essential for the safety of individuals with GSD Ia/b and for maintaining metabolic control. Continuous glucose monitoring (CGM) devices are also being used increasingly in the field of hepatic glycogen storage disorders and have been shown to be a valuable tool to assess glycemic control, make dietary changes, and improve metabolic control [11]. Table 10 presents the types of devices that are used for FSBG and CGM. Most clinics (20/21, 95%) recommended using CGM devices, and the patient’s medical insurance often dictated which brand was used.

Table 11 indicates which team members were involved in educating families on the use of CGM devices and in managing blood glucose levels. Table 11 also highlights the barriers to using CGM devices. Not listed are hesitation for use in very young children (2/21, 10%), difficulty with technological aspects, including sharing data with clinics (3/21, 14%), and the anxiety of parents leading to reactive treatment (1/21, 5%). Two of twenty-one respondents (10%) reported no barriers to using CGM systems.

Major determinants guiding the frequency of FSBG monitoring by clinics were new vs. previously diagnosed/established patients and whether the patient had a CGM device. In newly diagnosed patients, FSBG testing was recommended more frequently: before every feeding and/or UCCS dose and with symptoms of hypoglycemia. In the case of established and stable patients, the recommended frequency of FSBG checks were generally lower and varied from before every UCCS dose, at bedtime, 2–4 times/day, only based on symptoms of hypoglycemia, activity level, with dietary changes, and only every 6 months before clinic visits. For patients using CGM, clinics generally recommended FSBG checks only with low or high glucose alerts with abnormal-appearing CGM readings and just before clinic visits. One out of twenty-one clinics (5%) recommended no routine home FSBG monitoring.

Lactic acidosis is commonly seen in GSD Ia/b, particularly with sub-optimal metabolic control. The measurement of lactic acid is performed in a clinical setting. Although there may be some advantage to testing lactate levels at home to monitor impending hypoglycemia, presently, there is no FDA-approved lactate meter for medical or personal use. Because of this, none of the clinics recommended monitoring lactate levels at home.

### 3.9. Metabolic Control

Current published guidelines recommend the routine surveillance of young children with GSD Ia/b, including close monitoring of growth parameters and nutritional status, routine lab monitoring, and liver imaging [1,3]. Few publications provide age-specific guidelines for GSD Ia/b surveillance monitoring [6]. The survey respondents agreed with the need for comprehensive outpatient assessment and lab monitoring to achieve optimal glycemic and metabolic control, but the frequency varied (a minimum of every 6 months, every 2–4 months in other clinics). Some clinics utilized follow-up phone calls with GSD dietitians between outpatient appointments, and some clinics admitted patients when formulas were changed or Glycosade^®^ was initiated. Many clinics utilized CGM data as an adjunctive tool for the assessment of metabolic status, and two clinics (10%) offered annual inpatient hospitalization for in-depth hourly assessment of glucose and lactic acid, but this was not a common practice.

### 3.10. Hypoglycemia Management

Children with GSD Ia/b may experience hypoglycemia despite good adherence to their nutrition and UCCS plan, and it is crucial that caregivers know how to respond in such circumstances. The ACMG practice guidelines recommend treatment for blood glucose < 60 mg/dL with a quick-acting source of glucose, followed by a snack or UCCS to sustain normal blood glucose levels [1]. They recommend calculating the glucose dose on the basis on the desired glucose delivery rate [1]. There was no standard approach or clear consensus regarding treatment for GSD Ia/b mediated hypoglycemia among survey respondents, but most clinics recommended the twofold approach described in the ACMG guidelines, utilizing both a fast (glucose gel/tabs, Pixy Stix^®^, Smarties^®^) and a longer-acting carbohydrate source (UCCS, carbohydrate snack, formula) [1]. Some clinics provided detailed algorithms for hypoglycemia management based on FSBG measurements. Most clinics recommended retesting FSBG 10–15 min after the rescue intervention and repeating treatment if necessary. One clinic recommended fruit juice to correct hypoglycemia, which is contraindicated in GSD Ia/b because it contains fructose.

## 4. Limitations

It is important to consider that GSD Ia/b has an overall incidence of 1 in 100,000 [1]. The limitations of the survey included a small sample size, a lack of international data, a lack of data on growth outcomes, the exclusion of empagliflozin management, and the absence of statistical data. Additionally, the authors further limited the potential number of respondents by restricting the study population to those who cared for individuals with GSD Ia/b under 5 years of age. The survey was limited to North American respondents who participated in the metabolic dietitian listserv and/or were part of the Genetic Metabolic Dietitians International Membership. As such, we likely missed some practitioners who manage GSD Ia/b patients but were not members of these organizations. Additionally, the authors were unable to determine the response rate. As the purpose of this survey was to determine the current practice of medical nutrition therapy in this population, questions were limited to nutrition management and the use of empagliflozin, and other outcome measures, such as growth, were excluded.

## 5. Discussion

To the authors’ knowledge, this is the first study to assess the actual practice of dietitians regarding the nutrition management of children <5 years old with GSD Ia/b in North America. Current guidelines do not provide a definitive set of recommendations for managing very young children; the recommendations vary, and there have been no additional publications that provide detailed guidance in the management of young children. The survey results identified variations in nutrition management amongst clinics; however, there were key areas in which a majority consensus was seen (formula selection; age at cornstarch initiation; and the need for feeding therapy, GT feedings, and CGM devices).

Formula selection is important in this population. The ACMG guidelines recommend the use of a sucrose-free, soy-based infant formula, while the European guidelines recommend breastfeeding or lactose-free formula + maltodextrin [1,6]. Fructose, sucrose, and galactose are restricted sugars in GSD Ia/b, and most respondents utilized lactose/sucrose-free formulas; however, 24% of respondents reported the use of breastmilk, and the most abundant carbohydrate in mature breastmilk is lactose [12]. Despite the many nutritional, physical, and emotional benefits of breastmilk [13,14], ~50% of the carbohydrates are not utilizable in GSD Ia/b, and it is unknown whether the benefits outweigh the use of lactose. Additionally, 24% of respondents reported the use of sucrose-containing soy formulas. The long-term outcomes of allowing breastfeeding and sucrose-containing formulas are unknown. Because of the need for frequent feedings in this population, GT feedings can be lifesaving and were utilized in most surveyed clinics. However, there are inherent risks associated with the use of GT feedings for the young child with GSD Ia/b. These include pump malfunction and the increased risk of infection in those with GSD Ib.

The main goal of GSD Ia/b nutrition therapy is euglycemia; however, controversy remains regarding dietary carbohydrate and starch dosing to achieve this goal [2]. UCCS should be initiated slowly due to the inadequate amylase present when individuals are <2 years old, and most respondents followed the ACMG guidelines regarding initiating UCCS therapy between 6 and 12 months [1]. Both guidelines recommend feeding infants every 2–3 h during the day and suggest using continuous drip feeds overnight. Both guidelines recommend using the GIR to estimate continuous tube feeding rates and calculations using ideal body weight to estimate UCCS needs, but many clinics based their recommendations on estimated EGP [8]. Gluconeogenesis and glycogenolysis are the main processes contributing to EGP, and both processes are defective in GSD Ia/b [15]. Since the standardized equations used to determine EGP are based on fasting individuals, it is easy to overestimate glucose requirements in GSD Ia/b because of dietary carbohydrates and UCCS therapy. There were differences between surveyed clinics regarding recommendations for fasting tolerance and methods for assessing tolerance to the fasting period. Although Glycosade^®^ is not recommended for use in those <5 years old in the United States, approximately 25% of respondents reported its use in this population.

Infants normally begin eating solid food between 4 and 6 months of age. The timely introduction of solid food to compliment infant formula or breastmilk is important for providing a full range of nutrients for normal growth and development, exposing patients to various food textures and tastes, and promoting oral motor exercise to acquire and advance feeding skills. In the long term, this helps develop healthy eating behaviors and opportunities for social exchange. The goal to introduce solid food to infants with GSD Ia/b is no different. However, this needs to be carried out within the constraints of the sucrose-, fructose-, and lactose-restricted diet, with frequent feedings and cornstarch to maintain euglycemia. This creates its own unique feeding challenges and the need for interventions when there is a disruption or delay in meeting the above goals.

Because of the restrictive nature of the GSD Ia/b diet, vitamin supplementation is often necessary. According to the findings of our survey, the type, timing, and reason for nutritional supplementation was variable. Dysbiosis resulting in low intestinal microbial diversity in comparison with healthy controls has been studied in hepatic GSDs and is associated with an increased incidence of obesity, liver disease, and inflammatory bowel disease (particularly in GSD Ib) [16]. The main reason for these differences is unknown but has been attributed to the disease and/or the macro- and micronutrient content of the diet and the use of UCCS. The use of commercially formulated probiotics to overcome or manipulate dysbiosis is frequently recommended, but more research is needed regarding its efficacy, particularly in children <5 years old.

FSBG monitors and CGM devices are essential in evaluating the success of nutritional therapy for glycemic control. The accuracy of commercially available glucometers varies widely and depends on many factors, including operator technique; environmental factorsm such as altitude, temperature and humidity; the test strip enzyme (e.g., glucose oxidase vs. glucose dehydrogenase); and patient factors, such as hematocrit, blood lipid, pH level, etc. A reliable glucometer that overcomes most of the above challenges should be used for FSBG monitoring in GSD Ia/b. While no published studies comparing different glucometers in GSD Ia/b are available, the experience of one large GSD clinic was that the Freestyle Lite^®^ glucometer was best suited for GSD Ia/b because it showed the lowest interference by metabolites such as lactic acid. CGM systems are being used increasingly in the management of glycogen storage disorders, and almost all the respondents noted that CGM devices were used in their practice; however, over half noted that there were issues with obtaining insurance coverage.

## 6. Conclusions

The authors found a lack of evidence-based practice regarding estimating carbohydrate needs in this age group. In this survey, both EGP and GIR were used to help guide the carbohydrate content of the diet and UCCS dosing, and consensus is needed regarding best practices for estimating carbohydrate and UCCS requirements. There was no consistent “safe” fasting period amongst age groups. This is consistent with what the authors have experienced in their own practice. Everyone with GSD Ia/b is unique in their fasting tolerance, and genotype likely plays a role; more data are needed. Further data are also needed on the use of Glycosade^®^ and CGM in this age group. Additionally, recommendations for monitoring metabolic control and hypoglycemia management are needed. It is the authors’ hope that this survey provides initial insight into the challenges and variability of nutritional practices in this age group and leads to development of updated evidence-based guidelines that can be used as a standard of care by all practitioners.

## Figures and Tables

**Table 1:** ACMG and European nutrition management guidelines [1,6].

Category	ACMG [1]	European [6]
Formula	Infancy: soy-based, sugar-free formula or sucrose-, fructose-, and lactose-free formula Older children: sucrose-, fructose-, and lactose-free formula or elemental formula with a higher percentage of CHO	Infancy (0–12 months): breastfeeding/lactose-free formula 6–12 months: maltodextrin in formula feeding replaced by rice/corn (up to 6%)
Infant Feeding Interval	Every 2–3 h	Every 2–3 h
Nighttime feeds	When sleeping > 3–4 h: wake every 3–4 h, monitor BG, and offer feedings or use OGFs	0–12 months: OGFs or frequent feedings 1–3 years: OGFs or UCCS every 4 h 3–6 years: OGFs or UCCS every 4–6 h
Rate of continuous feeds	Infancy: 8–10 mg glucose/kg/min Older children: 4–8 mg glucose/kg/min	0–12 months: 7–9 mg glucose/kg/min 1–3 years: 6–8 mg glucose/kg/min 3–6 years: 6–7 mg glucose/kg/min
Start/ discontinuation of overnight feeds	Feed immediately after disconnecting or feed first and wait 30 min before disconnecting	Start within 1 h of the last meal, otherwise give a small oral or bolus feed Within 15 min after discontinuation, a feed should be given
Other GT	Risk with type Ib	Contraindicated with type Ib
Solid food introduction	Start at 4–6 months with infant cereals, followed by vegetables and then meat Limit or omit fruit, juice, and other sucrose-, fructose-, and lactose-containing foods Normal feeding progression with spoon feeding, drinking from a cup, and the introduction of table foods	
Cornstarch introduction	No consensus regarding cornstarch initiation, often trialed at 6 months to 1 year Start with a small dose and gradually increase to improve tolerance	>1 year Starting dose of 0.25 g/kg BW; increase dose slowly
Uncooked cornstarch dose	Young children: 1.6 g CS per kg BW (IBW) every 3–4 h Older children: 1.7–2.5 g CS per kg BW every 4–5 h, sometimes 6 h Ideally measured on a gram scale, otherwise with a tablespoon Additional guidance re: brand and mixing	1–3 years: 1–1.5 g/kg/dose every 4 h 3–6 years: 1.5–2 g/kg/dose every 4–6 h
Caloric distribution	CHO: 60–70%, high complex CHO PRO: 10–15% (for DRI) FAT: remaining calories (<30% if >2 years)	CHO: 60–65% PRO: 10–15% FAT: remaining calories (preferably vegetable oils with high linoleic acid)
Restriction/ elimination	No consensus regarding restriction of sucrose (fructose and glucose) or lactose (galactose and glucose), but often limited or avoided	Lactose, fructose, and sucrose restricted except for fruits, vegetables, and (small amounts of) milk products
Multivitamin/ mineral supplementation	Complete multivitamin with minerals Sugar-free soy-based milk fortified with calcium and vitamin D or calcium and vitamin D supplements	Follow dietary plan to provide essential nutrients, assess calcium and vitamin D intake, adequate vitamin B1, iron supplementation if needed

Abbreviations: *BW*—body weight, *CHO*—carbohydrate, *GT*—gastrostomy tube, *IBW*—ideal body weight, *OGFs*—overnight gastric feedings, *PRO*—protein, *UCCS*—uncooked cornstarch.

**Table 2:** Description of care team.

Team Member	Number of Clinics	Years of Experience
Dietitian	21	>10 years: 7 5–10 years: 10 1–5 years: 4
Physician	20	Not asked
Nurse practitioner Physician assistant	8	
Genetic counselor	11	
Nurse/case manager	6	
Support staff	8	

**Table 3:** Formula selection.

Formula Selection	Number of Clinics
Infant formula	
Sucrose, lactose free (Prosobee^®^)	17/21 (81%)
Hypoallergenic, sucrose, lactose-free (Nutramigen^®^)	9/21 (43%)
Hypoallergenic, amino acid-based, lactose-free (Alfamino^®^)	7/21 (33%)
Formula with sucrose (Isomil^®^)	5/21 (24%)
Breast feeding not recommended	16/21 (76%)
Children (1–5 years of age)	
Elemental, high-carbohydrate, 2% fat (Tolerex^®^)	9/21 (43%)
Peptide-based (PediaSure Peptide Unflavored ^®^)	7/21 (33%)
Elemental (Vivonex Pediatric^®^)	6/21 (29%)

**Table 4:** Clinicians’ experience/observation on tolerable fasting periods in individuals <5 years old with GSD Ia/b.

Time-Interval	<12 Months	13–24 Months	25–36 Months	37–48 Months	49–60 Months
1 h	3	0	0	0	0
1–1.5 h	1	1	1	0	0
1.5–2 h	15	7	2	1	0
2–2.5 h	5	9	10	6	3
2.5–3 h	1	4	9	12	14
Other	1	1	1	3	5

**Table 5:** Initiating UCCS.

Age of Introduction	Number of Clinics
6–9 months	7/21 (33%)
9–12 months	9/21 (43%)
>12 months	5/21 (24%)

**Table 6:** Glycosade^®^ use.

Time of Day	Number of Clinics
Nighttime	6/21 (29%)
Daytime	5/21 (24%)

**Table 7:** Introduction to solids.

Food Group	Number of Clinics
Vegetables	19/21 (90%)
Meats	10/21 (48%)
Cereals	5/21 (24%)

**Table 8:** Barriers to oral feeding.

Barrier	Number of Clinics
Lack of Interest	19/21 (90%)
Gagging	12/21 (57%)
Oral motor dysfunction	8/21 (38%)

**Table 9:** Supplementation use.

* Multivitamins	Number of Clinics
Meet deficiencies	10/19 (53%)
Case-by-case	3/19 (16%)
Routinely prescribed	2/19 (11%)
**Probiotics**	
GSD Ia	4/21 (19%)
GSD Ib	10/21 (48%)

* Two clinics (10%) did not provide a response.

**Table 10:** Device monitoring.

Finger Stick BG Monitoring	Number of Clinics
Freestyle Lite©	14/21 (67%)
**Continuous Glucose Monitoring**	
Dexcom©	13/21 (62%)
Libre©	6/21 (29%)

**Table 11:** CGM education/barriers.

CGM Education	Number of Clinics
Physician	9/21 (43%)
NP/PA	7/21 (33%)
RD	8/21 (38%)
RN	3/21 (14%)
Endocrine Staff	5/21 (24%)
Device Staff	2/21 (10%)
**Barriers**	
Lack of insurance coverage	10/21 (48%)
Lack of correlation	4/21 (19%)

## Data Availability

Data is contained within the article.

## Supplementary Materials

The following supporting information can be downloaded at: https://www.mdpi.com/article/10.3390/nu16193244/s1. Copy of the REDCap survey: Nutrition care in GSD types I a and b (for individuals aged 5 years and younger), Table S1: Survey of Nutrition Management of Children <5 years old with GSD I—main findings.
