# Supplementary material for: Nutrition Management in Children Less than 5 Years of Age with Glycogen Storage Disease Type I: Survey Results
Source: Nutrients. 2024 Sep 25;16(19):3244. doi: 10.3390/nu16193244 (PMC11478432; doi:10.3390/nu16193244)
Supplement: Supplementary file 1 [file nutrients-16-03244-s001.zip › nutrients-3146683-supplementary.pdf]

SUPPLEMENTARY TABLE:

Table S1: Survey of Nutrition Management of Children < 5 years with GSD I - Main findings

Nutrition Practice	# of clinics
Choice of infant feeding	
Sucrose, lactose free	17/21 (81%)
Formula with sucrose	5/21 (24%)
Breast feeding not allowed	16/21 (76%)
Tube Feedings Usage	
G tube feeding in 100% of patients	7/21 (33%)
G tube feeding in >50% patients	9/21 (43%)
Overnight bedwetting alarms	9/21 (43%)
Estimation of glucose/carbohydrate requirements	
Based on endogenous glucose production rate (Bier et al 1977)	12/21 (57%)
Based on needed glucose infusion rate	10/21 (48%)
Use of UCCS	
Age of introduction 6-9 months	7/21 (33%)
9-12 months	9/21 (43%)
After 12 months	5/21 (24%)
Brand of UCCS	ARGO®: 18/21 (86%)
Use of pancreatic enzymes to improve UCCS tolerance	5/21 (24%)
Use of Glycosade®	
In patients < 5 yrs	6/21 (29%)
Trial done with inpatient admission	10/21 (48%)
Trial done outpatient	8/21 (38%)
Introduction of solids	
Most common order of "first foods" recommended by clinics	Vegetables, meats, cereal
*Recommendation for amount of carbohydrates and/or sugars at solids introduction	16/19 (84%)
*No recommendation for amount of carbohydrates and sugars at solids introduction	5/19 (26%)
Referral for feeding therapy for patients	18/21 (86%)
Use of Nutritional Supplements	
*All patients getting MVI	2/19 (11%)
*Patients getting MVI based on volume of formula intake	10/19 (53%)
*MVI recommendation based on diet analysis, lab results	3/19 (16%)
Probiotic use	GSD Ia: 4/21 (19%),<br>GSD Ib: 10/21 (48%)
Blood glucose Monitoring	
Use of Freestyle Lite© glucometer	14/21 (67%)
Recommendation of continuous glucose monitoring	20/21 (95%)

*2 clinics (10%) did not provide a response.
